# Supplementary material for: Accounting for albedo change to identify climate-positive tree cover restoration
Source: Nat Commun. 2024 Mar 26;15:2275. doi: 10.1038/s41467-024-46577-1 (PMC10965905; doi:10.1038/s41467-024-46577-1)
Supplement: Supplementary file 1 — Supplementary Information [file 41467_2024_46577_MOESM1_ESM.pdf]

Supplementary Information

Table S1. IGBP Land Cover Classes Used in this Study International Geosphere-Biosphere Programme (IGBP) legend for the open, savanna and forest land cover classes used in this study, from the MODIS/ Terra+Aqua Land Cover Dataset, with class number in parentheses next to the class name[1,2].

**Name**	**Acronym**	**Description**
**Evergreen needleleaf forests (1)**	ENF	Lands dominated by needleleaf woody vegetation with a percent cover >60% and height exceeding 2 m. Almost all trees remain green all year. Canopy is never without green foliage.
**Evergreen broadleaf forests (2)**	EBF	Lands dominated by broadleaf woody vegetation with a percent cover >60% and height exceeding 2 m. Almost all trees and shrubs remain green year-round. Canopy is never without green foliage.
**Deciduous needleleaf forests (3)**	DNF	Lands dominated by woody vegetation with a percent cover >60% and height exceeding 2 m. Consists of seasonal needleleaf tree communities with an annual cycle of leaf-on and leaf-off periods.
**Deciduous broadleaf forests (4)**	DBF	Lands dominated by woody vegetation with a percent cover >60% and height exceeding 2 m. Consists of broadleaf tree communities with an annual cycle of leaf-on and leaf-off periods.
**Mixed forests (5)**	MF	Lands dominated by trees with a percent cover >60% and height exceeding 2 m. Consists of tree communities with interspersed mixtures or mosaics of the other four forest types. None of the forest types exceeds 60% of landscape.
**Woody savannas (8)**	WSA	Lands with herbaceous and other understory systems, and with forest canopy cover between 30% and 60%. The forest cover height exceeds 2 m.
**Savannas (9)**	SAV	Lands with herbaceous and other understory systems, and with forest canopy cover between 10% and 30%. The forest cover height exceeds 2 m.
**Open shrublands (7)**	OSH	Lands with woody vegetation less than 2 m tall and with shrub canopy cover between 10% and 60%. The shrub foliage can be either evergreen or deciduous.
**Grasslands (10)**	GRA	Lands with herbaceous types of cover. Tree and shrub cover is less than 10%.
**Croplands (12)**	CRO	Lands covered with temporary crops followed by harvest and a bare soil period (e.g., single and multiple cropping systems). Note that perennial woody crops will be classified as the appropriate forest or shrub land cover type.
**Cropland/Natural Vegetation Mosaics (14)**	MOS	Mosaics of small-scale cultivation 40-60% with natural tree, shrub, or herbaceous vegetation.

Table S2: Area, carbon, and net climate impacts (NCI) across the total biome and places identified as an opportunity to restore tree cover: the Griscom opportunity map[3], the Bastin opportunity map[4], the Walker opportunity map[5], and unfiltered by an opportunity map (TotalBiome). To be comprehensive, we show all biomes for which we have data, but in the main results we focus on forest and woody savanna biomes that are suitable for upland trees and that represent larger areas. We also show the percentage of the area within each biome that experiences less than 50% albedo offset (AO), as well as overall Pg CO_2e and Mg CO_2e ha^{-1} for those areas, and the percentage of the area that is net climate-negative (i.e., >100% albedo offset). Uncertainties in square brackets reflect the maximum and minimum values across the six radiative kernels.

	**Area**	**Carbon only**	**Total NCI (carbon+albedo)**	**NCI average density**	**Mean Albedo Offset**	**<50%AO**	**<50%AO NCI**	**<50%AO NCI average rate**	**>100%AO**
**Units**	Mha	Pg CO_2e	Pg CO_2e	Mg CO_2e ha^{-1}	%	% area	Pg CO_2e	Mg CO_2e ha^{-1}	% area
**Tundra**									
Griscom	0	0	0 [0 - 0]	68 [44 - 96]	66 [78 - 53]	38 [38 - 38]	0 [0 - 0]	112 [95 - 125]	0 [41 - 0]
Bastin	53	7	-2 [-6 - 1]	-46 [-107 - 14]	136 [183 - 89]	11 [7 - 24]	1 [1 - 2]	150 [174 - 128]	63 [76 - 42]
Walker	198	26	-19 [-36 - -6]	-95 [-180 - -28]	174 [240 - 122]	6 [3 - 14]	2 [1 - 3]	133 [144 - 125]	76 [87 - 58]
TotalBiome	458	51	-50 [-87 - -19]	-110 [-190 - -41]	198 [269 - 136]	9 [5 - 18]	5 [3 - 11]	132 [138 - 129]	71 [81 - 57]
**Boreal Forests/Taiga**									
Griscom	3	1	1 [1 - 1]	242 [200 - 271]	37 [48 - 29]	76 [64 - 81]	1 [1 - 1]	321 [304 - 335]	11 [15 - 7]
Bastin	176	31	4 [-3 - 12]	24 [-20 - 66]	86 [111 - 62]	27 [18 - 41]	8 [5 - 11]	159 [161 - 154]	39 [53 - 23]
Walker	144	23	-9 [-18 - 1]	-59 [-125 - 6]	137 [178 - 96]	14 [9 - 22]	3 [2 - 5]	177 [181 - 167]	63 [75 - 45]
TotalBiome	1411	289	60 [-1 - 120]	42 [0 - 85]	79 [100 - 58]	28 [17 - 45]	70 [42 - 109]	176 [175 - 173]	34 [46 - 19]
**Temperate Grasslands, Savannas & Shrublands**									
Griscom	32	9	5 [4 - 6]	149 [121 - 176]	48 [58 - 39]	58 [46 - 69]	4 [4 - 5]	241 [240 - 243]	14 [19 - 9]
Bastin	99	14	-4 [-8 - -1]	-45 [-82 - -6]	132 [159 - 104]	22 [18 - 27]	4 [3 - 5]	196 [199 - 194]	62 [66 - 56]
Walker	85	13	-4 [-8 - -1]	-48 [-88 - -6]	133 [160 - 104]	19 [14 - 25]	3 [2 - 4]	193 [194 - 191]	63 [68 - 56]
TotalBiome	1039	107	-102 [-144 - -58]	-98 [-138 - -56]	195 [235 - 155]	17 [14 - 22]	35 [29 - 44]	198 [200 - 194]	72 [75 - 67]
**Mediterranean Forests, Woodlands & Scrub**									
Griscom	56	14	5 [4 - 6]	95 [64 - 115]	63 [75 - 56]	43 [36 - 49]	6 [5 - 7]	254 [252 - 254]	26 [32 - 23]
Bastin	20	5	2 [1 - 2]	81 [53 - 100]	66 [78 - 58]	42 [35 - 48]	2 [2 - 2]	237 [232 - 239]	30 [35 - 27]
Walker	21	5	0 [-1 - 1]	2 [-45 - 29]	99 [119 - 88]	25 [21 - 29]	1 [1 - 1]	246 [245 - 246]	46 [54 - 42]
TotalBiome	326	52	-30 [-44 - -21]	-92 [-135 - -65]	158 [185 - 141]	24 [20 - 27]	19 [15 - 21]	241 [237 - 242]	60 [63 - 57]

	Area	Carbon only	Total NCI (carbon+albedo)	NCI average density	Mean Albedo Offset	<50%AO	<50%AO NCI	<50%AO NCI average rate	>100%AO
**Temperate Conifer Forests**									
Griscom	26	10	6 [5 - 7]	238 [200 - 273]	37 [47 - 27]	71 [62 - 78]	7 [6 - 7]	353 [354 - 357]	15 [18 - 12]
Bastin	30	8	3 [2 - 5]	116 [73 - 153]	59 [74 - 46]	49 [38 - 60]	4 [3 - 5]	278 [286 - 274]	27 [33 - 21]
Walker	37	10	2 [0 - 4]	56 [0 - 98]	79 [100 - 63]	35 [27 - 43]	4 [3 - 5]	302 [314 - 293]	41 [49 - 34]
TotalBiome	370	126	58 [41 - 73]	156 [110 - 196]	54 [68 - 42]	50 [41 - 62]	69 [59 - 81]	372 [387 - 357]	26 [31 - 21]
**Temperate Broadleaf & Mixed Forests**									
Griscom	242	77	54 [47 - 60]	223 [194 - 246]	30 [39 - 23]	80 [67 - 89]	51 [42 - 58]	264 [260 - 272]	5 [7 - 3]
Bastin	111	35	23 [19 - 25]	203 [174 - 227]	35 [44 - 27]	75 [64 - 83]	22 [19 - 25]	267 [262 - 272]	9 [11 - 6]
Walker	71	21	12 [9 - 14]	164 [129 - 194]	44 [56 - 34]	65 [52 - 75]	12 [10 - 14]	261 [258 - 267]	17 [20 - 13]
TotalBiome	1215	367	221 [179 - 254]	182 [147 - 209]	40 [51 - 31]	69 [57 - 78]	227 [185 - 262]	270 [267 - 276]	13 [17 - 10]
**Tropical & Subtropical Grasslands, Savannas & Shrublands**									
Griscom	49	16	13 [13 - 14]	271 [258 - 282]	18 [22 - 15]	77 [73 - 81]	13 [13 - 14]	347 [351 - 341]	8 [12 - 6]
Bastin	242	61	39 [33 - 43]	163 [138 - 177]	35 [45 - 29]	70 [64 - 74]	44 [41 - 47]	262 [262 - 262]	17 [21 - 14]
Walker	147	34	23 [19 - 24]	153 [132 - 165]	33 [42 - 28]	67 [61 - 72]	24 [22 - 25]	241 [247 - 237]	15 [21 - 12]
TotalBiome	2117	471	83 [-31 - 148]	39 [-15 - 70]	82 [107 - 69]	54 [50 - 57]	365 [339 - 381]	320 [323 - 319]	38 [40 - 36]
**Tropical & Subtropical Coniferous Forests**									
Griscom	13	5	4 [4 - 4]	318 [306 - 325]	15 [18 - 13]	90 [86 - 93]	4 [4 - 4]	345 [345 - 345]	1 [2 - 1]
Bastin	7	2	2 [1 - 2]	224 [210 - 234]	25 [30 - 22]	75 [70 - 79]	2 [1 - 2]	302 [304 - 301]	13 [15 - 11]
Walker	5	1	1 [1 - 1]	166 [150 - 179]	35 [42 - 31]	61 [56 - 66]	1 [1 - 1]	274 [278 - 271]	19 [24 - 16]
TotalBiome	68	22	17 [16 - 18]	250 [235 - 260]	24 [28 - 21]	75 [71 - 78]	17 [16 - 18]	339 [339 - 339]	13 [15 - 12]
**Tropical & Subtropical Dry Broadleaf Forests**									
Griscom	73	26	21 [20 - 22]	292 [278 - 300]	17 [21 - 14]	82 [76 - 86]	21 [20 - 22]	351 [358 - 348]	6 [8 - 5]
Bastin	37	11	8 [7 - 8]	216 [199 - 226]	26 [32 - 22]	70 [65 - 73]	8 [8 - 9]	313 [316 - 312]	16 [20 - 14]
Walker	38	10	7 [6 - 7]	185 [161 - 199]	33 [41 - 28]	61 [53 - 67]	7 [6 - 7]	291 [303 - 283]	14 [22 - 10]
TotalBiome	381	111	77 [68 - 82]	203 [179 - 216]	30 [38 - 26]	62 [56 - 66]	81 [76 - 85]	345 [355 - 338]	21 [26 - 19]
**Tropical & Subtropical Moist Broadleaf Forests**									
Griscom	316	154	140 [136 - 143]	443 [432 - 451]	9 [11 - 7]	94 [90 - 96]	139 [135 - 142]	470 [473 - 469]	1 [2 - 1]
Bastin	75	30	26 [25 - 27]	345 [329 - 358]	15 [19 - 12]	89 [83 - 94]	25 [24 - 27]	380 [384 - 380]	2 [4 - 1]
Walker	68	32	29 [28 - 29]	424 [410 - 434]	11 [14 - 9]	85 [80 - 89]	28 [27 - 29]	495 [507 - 486]	6 [8 - 4]

	Area	Carbon only	Total NCI (carbon+albedo)	NCI average density	Mean Albedo Offset	<50%AO	<50%AO NCI	<50%AO NCI average rate	>100%AO
TotalBiome	1918	1214	1148 [1127 - 1160]	598 [588 - 605]	5 [7 - 4]	94 [92 - 95]	1149 [1129 - 1161]	637 [637 - 636]	3 [4 - 2]
**Mangroves**									
Griscom	6	3	3 [3 - 3]	469 [458 - 476]	7 [9 - 5]	95 [93 - 96]	3 [3 - 3]	491 [487 - 494]	2 [2 - 2]
Bastin	3	1	1 [1 - 1]	370 [363 - 374]	7 [9 - 6]	94 [92 - 95]	1 [1 - 1]	391 [391 - 392]	2 [3 - 2]
Walker	1	1	1 [1 - 1]	408 [401 - 412]	8 [9 - 7]	89 [86 - 90]	1 [1 - 1]	457 [463 - 453]	4 [5 - 3]
TotalBiome	29	16	15 [15 - 15]	527 [519 - 532]	5 [6 - 4]	96 [94 - 96]	15 [15 - 15]	551 [550 - 553]	2 [3 - 2]
**Flooded Grasslands & Savannas**									
Griscom	6	2	1 [1 - 1]	128 [104 - 151]	51 [60 - 42]	42 [23 - 71]	0 [0 - 1]	183 [194 - 180]	5 [6 - 4]
Bastin	11	3	2 [2 - 2]	190 [178 - 199]	23 [28 - 20]	79 [73 - 87]	2 [2 - 2]	244 [250 - 236]	7 [8 - 7]
Walker	6	2	1 [1 - 1]	196 [182 - 208]	26 [31 - 22]	71 [63 - 82]	1 [1 - 1]	259 [273 - 247]	7 [9 - 6]
TotalBiome	109	22	10 [7 - 12]	91 [65 - 108]	55 [68 - 47]	62 [57 - 69]	17 [16 - 18]	245 [252 - 238]	24 [26 - 24]
**Montane Grasslands & Shrublands**									
Griscom	3	1	0 [0 - 0]	138 [115 - 154]	42 [51 - 35]	59 [54 - 63]	0 [0 - 0]	258 [263 - 253]	24 [29 - 19]
Bastin	14	3	1 [1 - 1]	77 [44 - 102]	64 [80 - 53]	54 [49 - 60]	2 [2 - 2]	240 [243 - 238]	31 [35 - 28]
Walker	45	7	-8 [-11 - -5]	-168 [-253 - -115]	216 [275 - 180]	19 [16 - 23]	2 [1 - 2]	202 [209 - 197]	70 [73 - 66]
TotalBiome	477	33	-111 [-150 - -90]	-233 [-315 - -188]	436 [554 - 371]	12 [10 - 13]	14 [13 - 16]	260 [267 - 252]	83 [85 - 82]
**Deserts & Xeric Shrublands**									
Griscom	1	0	0 [0 - 0]	93 [84 - 103]	40 [46 - 33]	61 [58 - 66]	0 [0 - 0]	205 [207 - 198]	28 [30 - 25]
Bastin	40	4	-3 [-5 - -2]	-81 [-114 - -52]	186 [221 - 155]	23 [21 - 26]	2 [1 - 2]	179 [182 - 175]	65 [68 - 62]
Walker	22	3	0 [-1 - 0]	-13 [-47 - 14]	109 [133 - 90]	28 [24 - 33]	1 [1 - 1]	182 [189 - 177]	51 [57 - 46]
TotalBiome	631	24	-129 [-157 - -108]	-205 [-250 - -171]	647 [767 - 556]	10 [9 - 11]	10 [9 - 11]	156 [160 - 154]	86 [87 - 85]
**Globally**									
Griscom	828	318	254 [238 - 267]	307 [287 - 323]	20 [25 - 16]	82 [74 - 87]	251 [232 - 265]	372 [379 - 369]	6 [8 - 5]
Bastin	916	214	101 [71 - 126]	110 [78 - 137]	53 [67 - 41]	52 [45 - 59]	127 [113 - 141]	266 [272 - 259]	29 [35 - 23]
Walker	889	186	35 [-10 - 72]	40 [-11 - 81]	81 [105 - 61]	35 [30 - 42]	90 [80 - 100]	285 [300 - 269]	46 [53 - 36]
TotalBiome	10548	2905	1266 [839 - 1587]	120 [80 - 150]	56 [71 - 45]	49 [44 - 55]	2095 [1945 - 2234]	405 [423 - 387]	37 [41 - 33]

Table S3: Proportion of maximum carbon storage offset by albedo in project-pixels. We show pixel counts and percentage of project pixels that fall into different albedo offset bins. The column on the right indicates only project-pixels that overlapped with at least one of the published opportunity maps[3–5]. When we report statistics in the main text, we assume that locations where our model does not predict an albedo offset (i.e., NA) would have a >100% albedo offset since these are generally in places like deserts where carbon accumulation is very low and the open lands are highly reflective.

Albedo offset range %	Overall pixel count (%)	Pixels overlapping opportunity map count (%)
NA	52,354 (6)	448,562 (55.0)
>100	74,788 (9)	306,121 (37.5)
100 to 90.1	12,509 (2)	5,209 (0.6)
90 to 80.1	16,734 (2)	5,099 (0.6)
80 to 70.1	21,891 (3)	5,044 (0.6)
70 to 60.1	26,369 (3)	5,022 (0.6)
60 to 50.1	33,131 (4)	4,649 (0.6)
50 to 40.1	49,481 (6)	4,195 (0.6)
40 to 30.1	105,687 (13)	3,916 (0.5)
30 to 20.1	148,026 (18)	3,416 (0.4)
20 to 10.1	143,048 (18)	2,830 (0.3)
10 to 0.1	82,012 (10)	2,575 (0.3)
<0	49,624 (6)	19,016 (2.3)

Figure S1. Most likely land cover maps. (a) Most likely open lands include open shrublands (OSH), grasslands (GRA), croplands (CRO) and cropland/natural vegetation mosaics (MOS), and b) most likely forest include evergreen needleleaf forests (ENF), evergreen broadleaf forests (EBF), deciduous needleleaf forests (DNF), deciduous broadleaf forests (DBF), mixed forests (MF) as well as woody savanna (WSA). These maps are based on IGBP-classified MODIS (MCD12Q1.06) land cover type global 500-m (years 2001 and 2010), reprojected on a global 0.005 * 0.005 lat/long WGS84 grid with nearest neighbors and expanded with neighborhood analysis (see Methods). We provide the spatial data for these (see Data Availability).

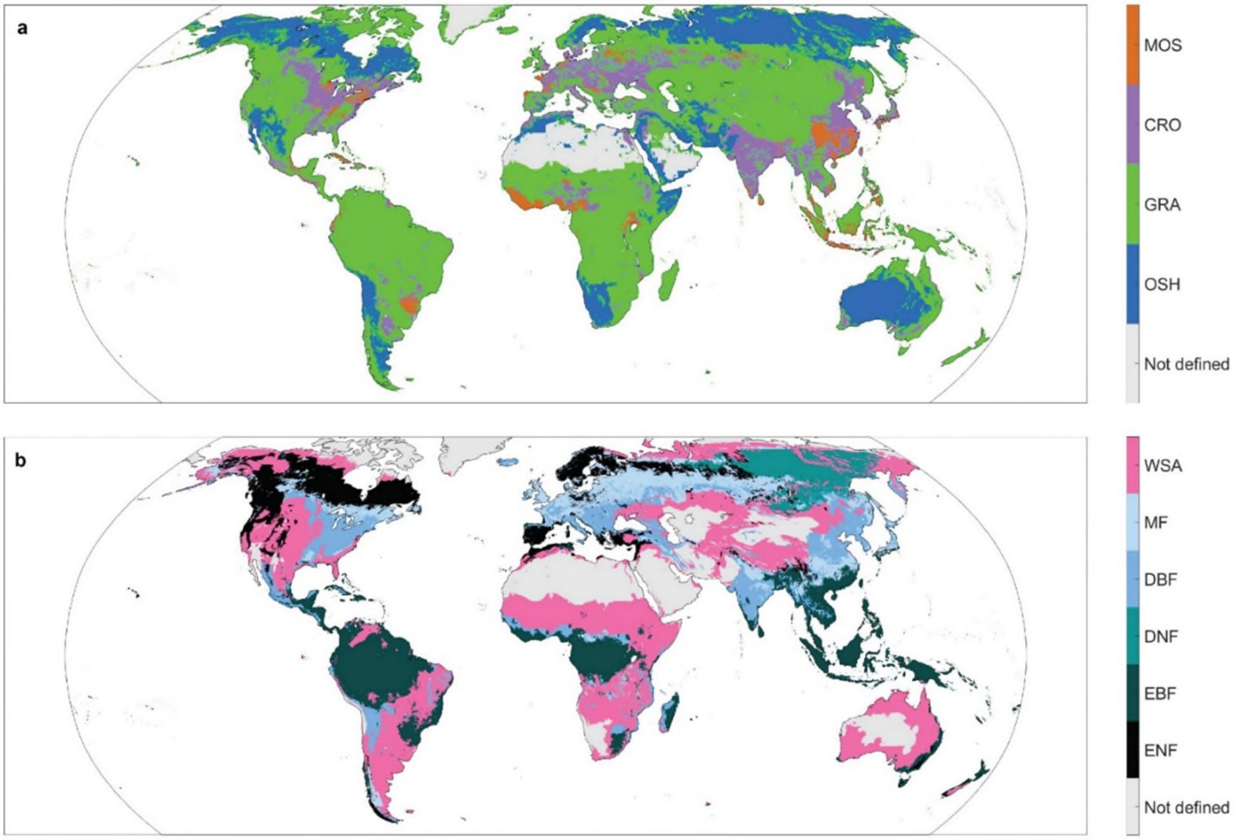

Figure S2. Global maps of albedo change and maximum potential carbon storage (in Mg CO_2e ha^{-1}) We use the same color scale as Figure 1a. (a) Potential albedo change from the most likely open land to woody savanna/forest cover transition, irrespective of actual opportunity or suitability for restoration of tree cover. We show and provide the median value across six radiative kernels[6–11], but also provide maximum and minimum values (see Data Availability). (b) Maximum carbon storage in above and belowground biomass from Walker et al[5]. In both maps the scale bar to the left of the color ramp indicates 5%, 10%, 25%, 50%, 75%, 90%, and 95% land-area percentiles (top to bottom) in Mg CO_2e ha^{-1}. For comparison to other studies, we also label the color ramp in units of Mg Ce ha^{-1} (italicized text on left side).

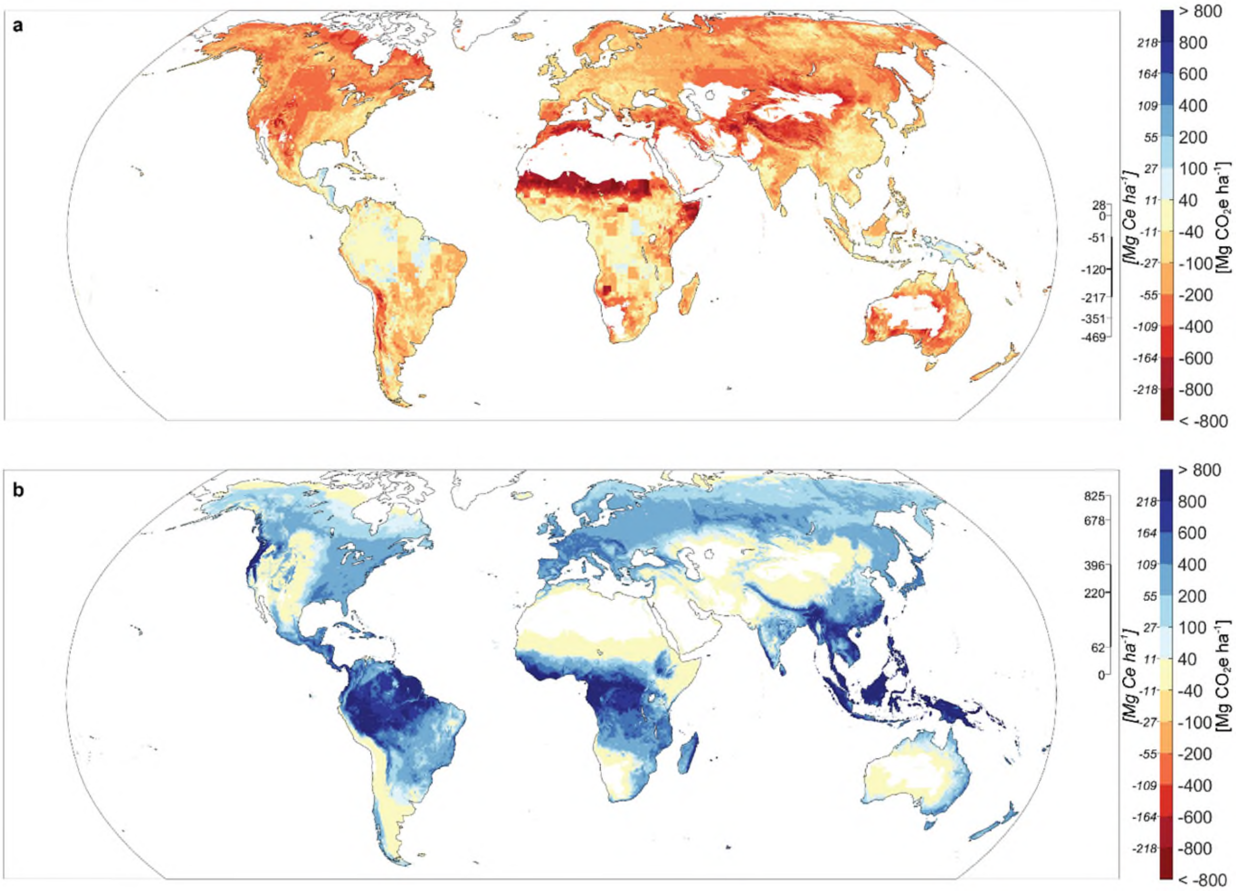

Figure S3. Proportion of project pixels with different net climate impacts (Mg CO_2e ha^{-1}). We also show the cumulative proportion of pixels (red line, right y-axis). This figure does not include NA values.

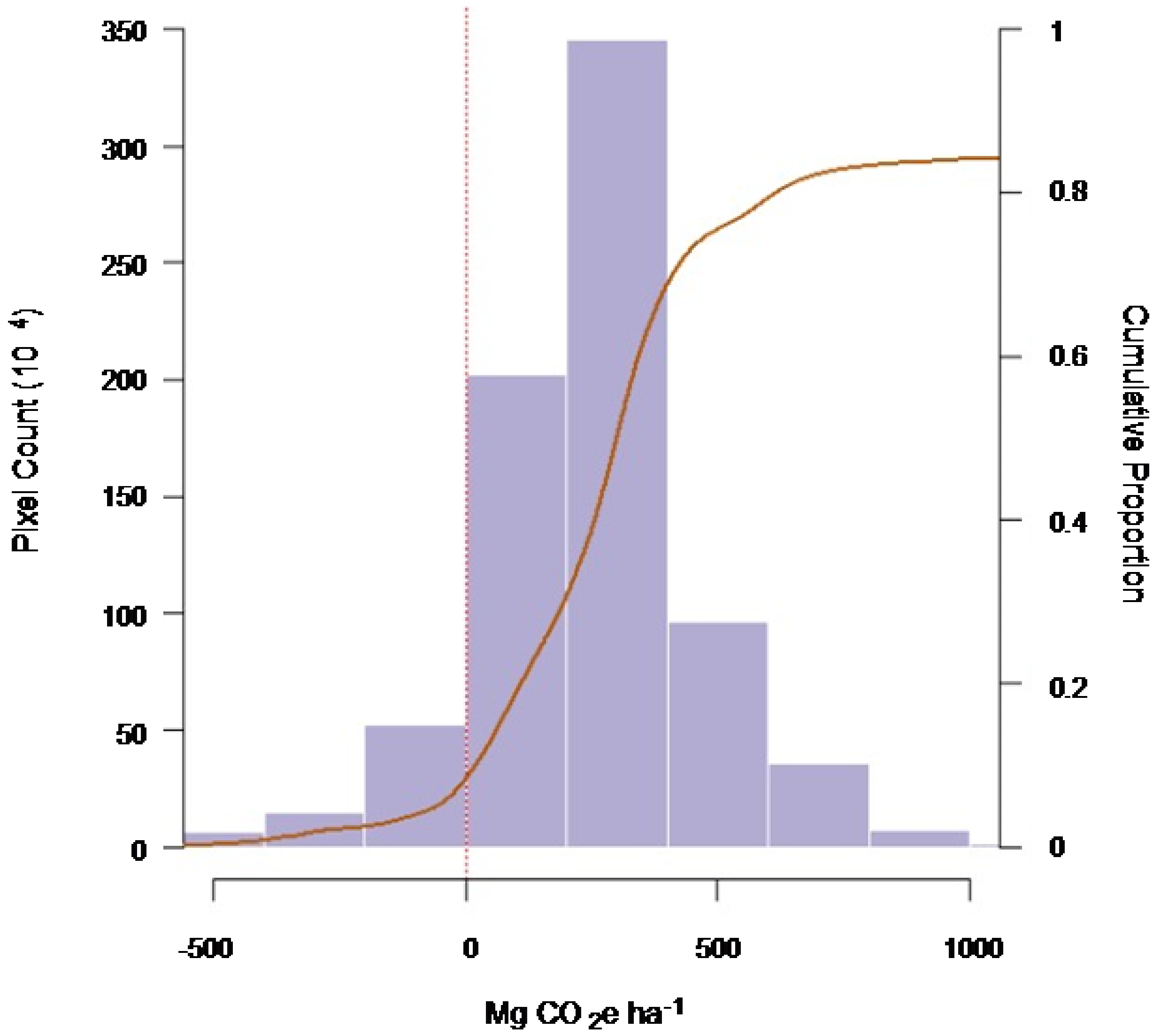

Figure S4. Distribution of on-the-ground projects. From past, on-going, and planned projects that are part of the Grain for Green Program[12] (large gray circles) or uploaded onto Restor[13] (black dots). Project locations are shown on top of the net-climate impact map (Fig. 1a).

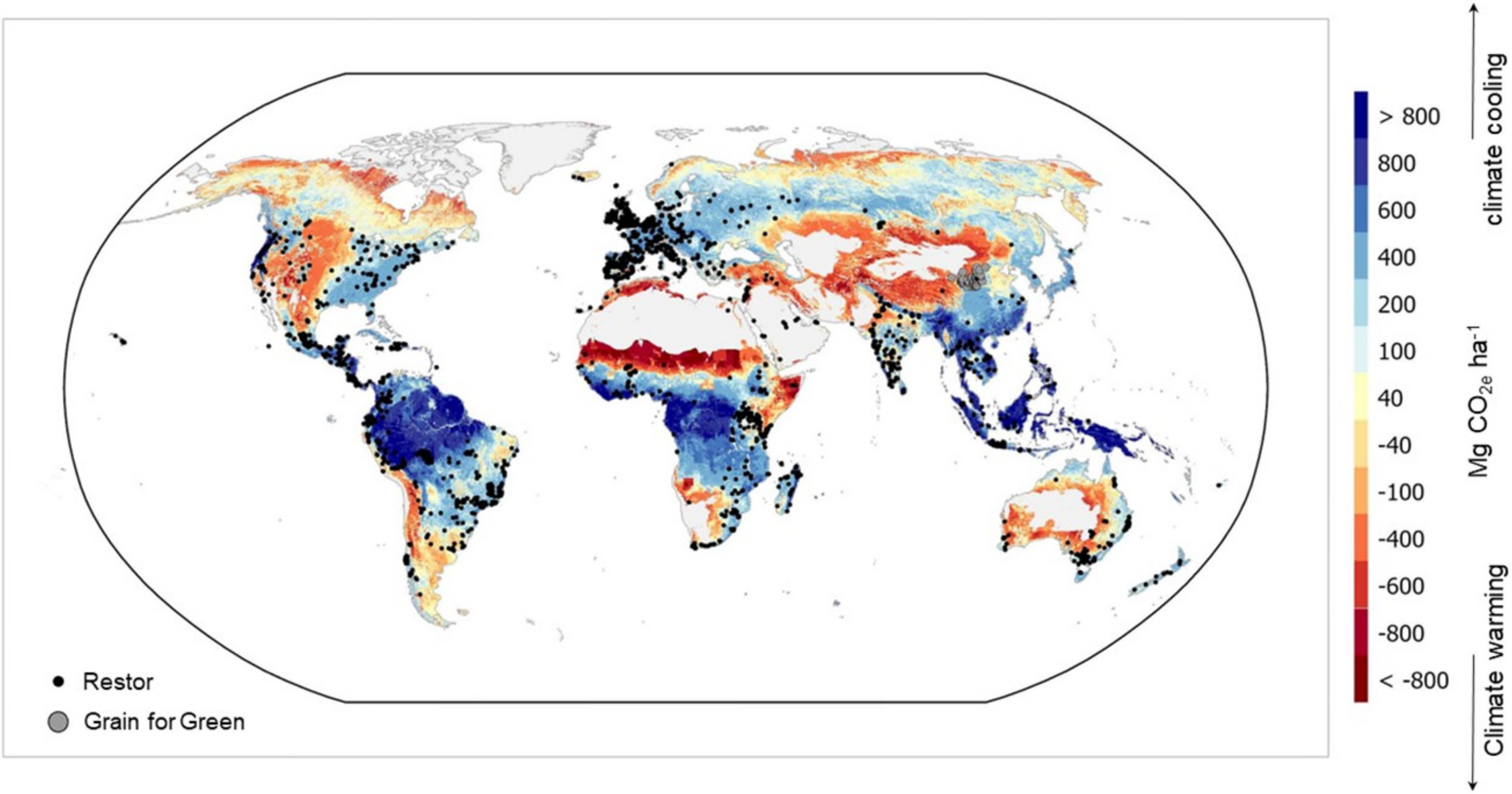

Figure S5. Areas experiencing a greater than 50% albedo offset (AO) based on the range observed across six radiative kernels. We identified the areas with a greater than 50% albedo offset using the minimum radiative forcing observed across all radiative kernels, the median value, and the maximum value. Some locations always have less than a 50% albedo offset (teal) or a greater than 50% offset (darkest brown), regardless of the kernel used. However, a few locations will transition to greater than or less than 50% offset depending on the kernel used. Brown indicates that pixels have greater than 50% offset in both median and maximum radiative forcing maps, while lightest brown indicate pixel that only shown substantial offset when considering maximum value. We provide the spatial data (see Data Availability).

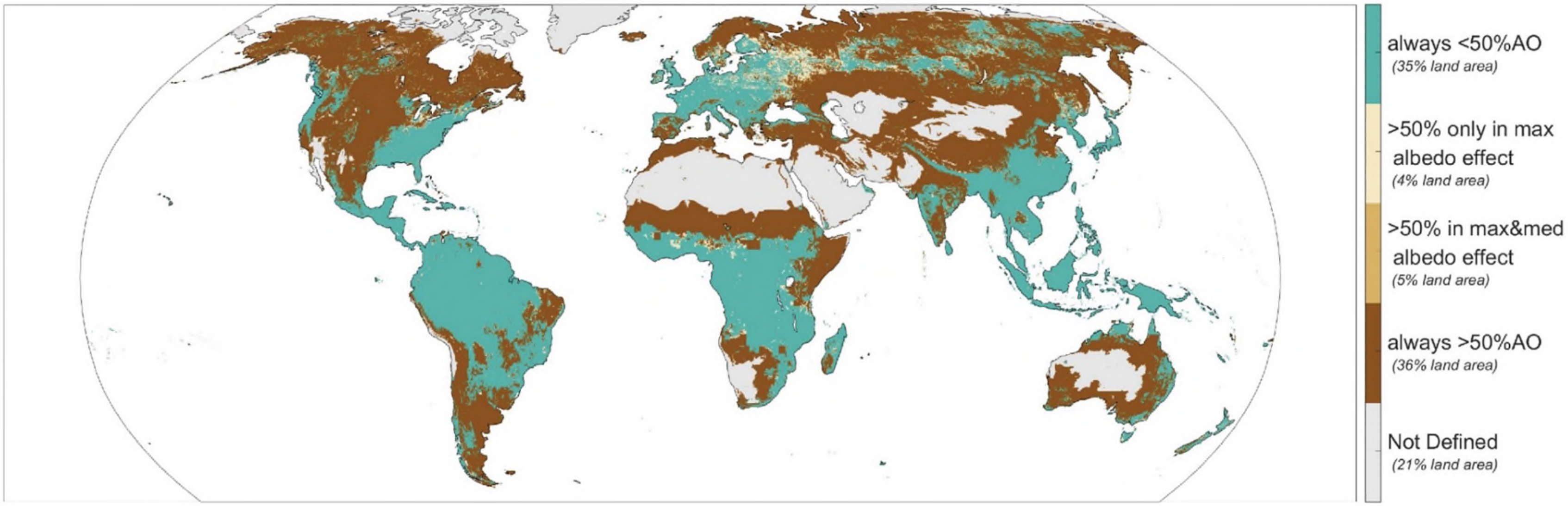

Figure S6. Net climate impact map with ESA-CCI. a) Net climate impact calculated with a version of Walker et al.[5] where the high values are truncated at the 85% value observed in a current biomass map (ESA-CCI)[14](“ESA-truncated Walker”, see Data Availability), b) absolute difference between ESA-truncated Walker and original Walker et al. net climate impact maps, and c) percent difference between both net climate impact maps.[5] Places with the greatest difference are those where the Walker map may either over-predict biomass (e.g., northern Russia) or where the current biomass may be lower than potential (e.g., China). The scale bar immediately to the right of the maps indicates the 5%, 10%, 25%, 50%, 75%, 90%, and 95% land-area percentiles (top to bottom).

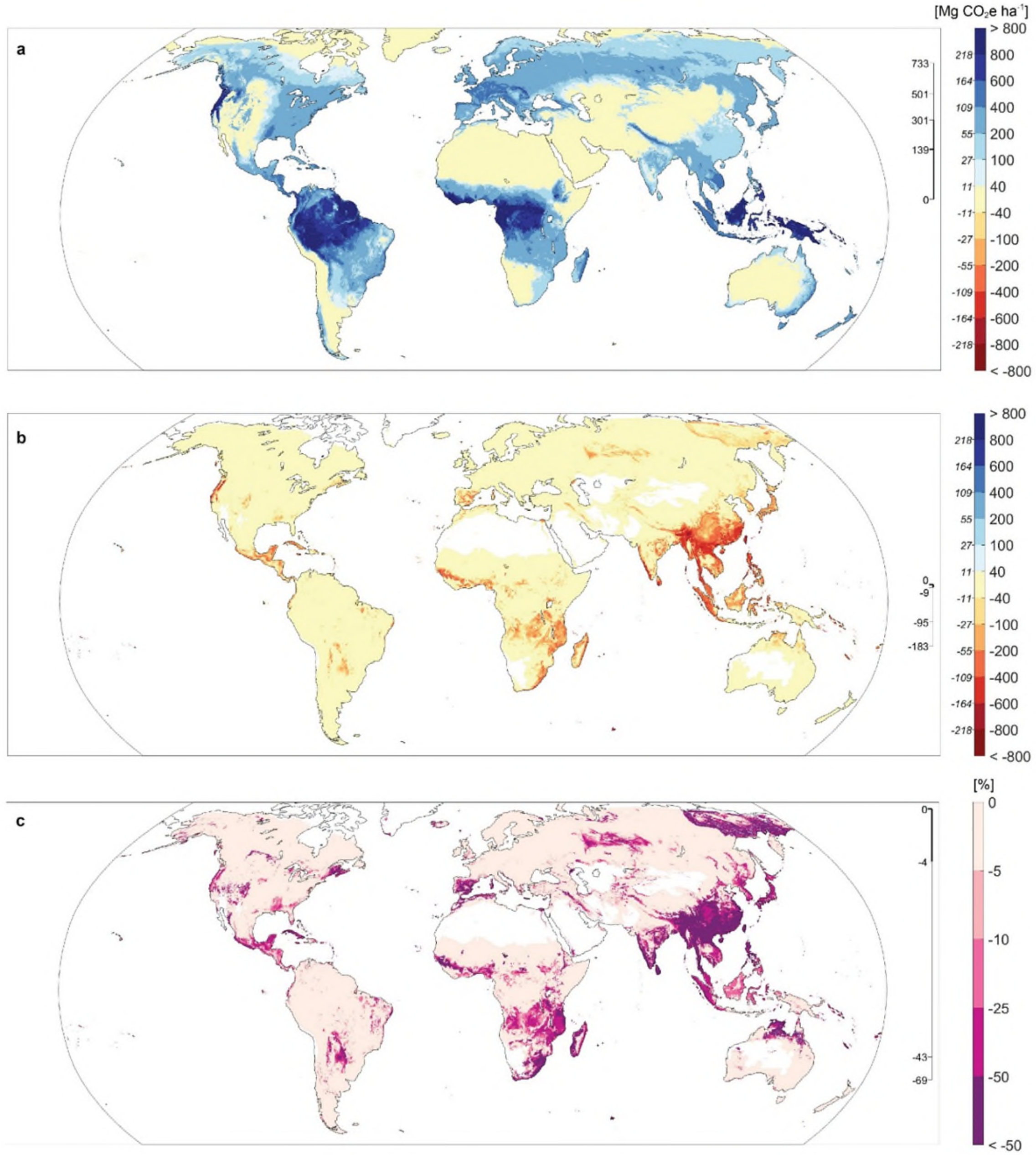

Figure S7. Different scenarios of albedo and carbon change through time. The combined, cumulative CO_2e flux for each is shown in the upper panel and the net climate effect of their combination is shown in the lower panel. Results are divided into six cases that vary the pace of carbon accumulation (fast = cases 1, 2, 3; slow = cases 4, 5, 6), and vary the albedo offset (100% = cases 1, 4; 50% = cases 2, 5; 10% = cases 3, 6). Within each case we also vary how soon albedo reaches its maximum before carbon. Ocean and land releases of CO_2 in response to carbon removals from tree cover restoration are also included in the simulations as an additional term, and this is included in the net cumulative CO_2e. We find that the magnitude of the net climate effect changes through time but the sign stays consistent in all but one of the twelve scenarios, with the one exception showing only a slight climate-positive effect before giving way to a climate-negative effect in the long-term.

Where the albedo offset is large (cases 1 and 4), the net climate impact is climate-negative (net cumulative CO_2e flux is positive indicating an effect equivalent to a release of CO_2 to the atmosphere) regardless of assumptions around time horizon. When the albedo response precedes the carbon response by a lot (50% earlier), we see an earlier warming response (net CO_2e emission). Even at longer time frames we see an enduring warming effect because the carbon that was removed from the atmosphere is compensated by ocean and land releases while the large albedo warming is sustained. When the carbon accumulation is faster, so too is the albedo approach to its maximum, and thus we find an earlier net climate warming response.

Where the albedo offset is small (cases 3 and 6), the net climate impact is consistently negative and is insensitive to the timing of albedo relative to carbon. In this situation, atmospheric carbon decreases to a minimum and then sees a rebound as ocean and land outgassing respond over time. We see a maximum climate cooling effect at around the time that the carbon removals saturate, followed by a slow decline as the ocean and land response kick in over time. When the carbon accumulation is faster, in this case we naturally find an earlier net climate cooling effect.

Where the albedo offset is intermediate (cases 2 and 4) we find the most complex temporal dynamics. The short-term effect (20 to 50 years) is a net warming when the albedo effect leads the carbon effect by a lot (50% earlier). However, the medium-term effect can be neutral, a modest warming, or even a slight cooling, as the carbon removals reach their maximum rate, outpacing compensation by ocean and land releases. The long-term effect (150 – 200 years) is consistently a net warming response of the climate system as the albedo effect persists but the carbon removals become compensated by the ocean and land release of CO_2.

Code for recreating these figures is available (see Code Availability).

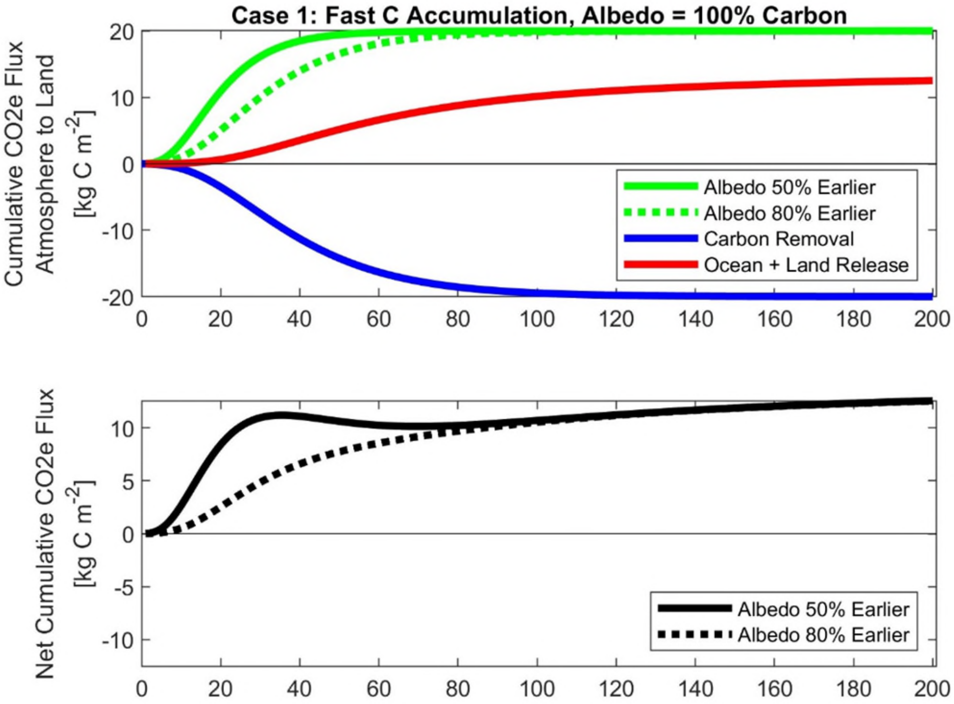

Case 1: Fast C Accumulation, Albedo = 100% Carbon
Cumulative CO2e Flux Atmosphere to Land [kg C m^-2]
Albedo 50% Earlier
Albedo 80% Earlier
Carbon Removal
Ocean + Land Release
Net Cumulative CO2e Flux [kg C m^-2]
Albedo 50% Earlier
Albedo 80% Earlier

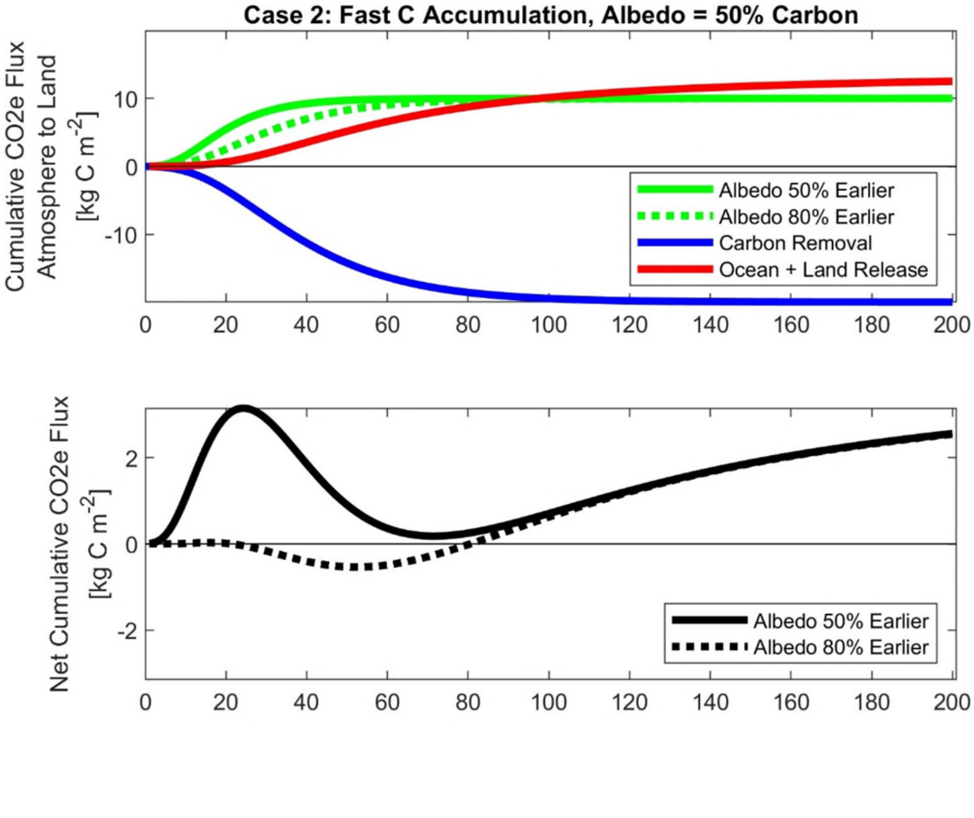

Case 2: Fast C Accumulation, Albedo = 50% Carbon
Cumulative CO2e Flux Atmosphere to Land [kg C m^-2]
Albedo 50% Earlier
Albedo 80% Earlier
Carbon Removal
Ocean + Land Release
Net Cumulative CO2e Flux [kg C m^-2]
Albedo 50% Earlier
Albedo 80% Earlier

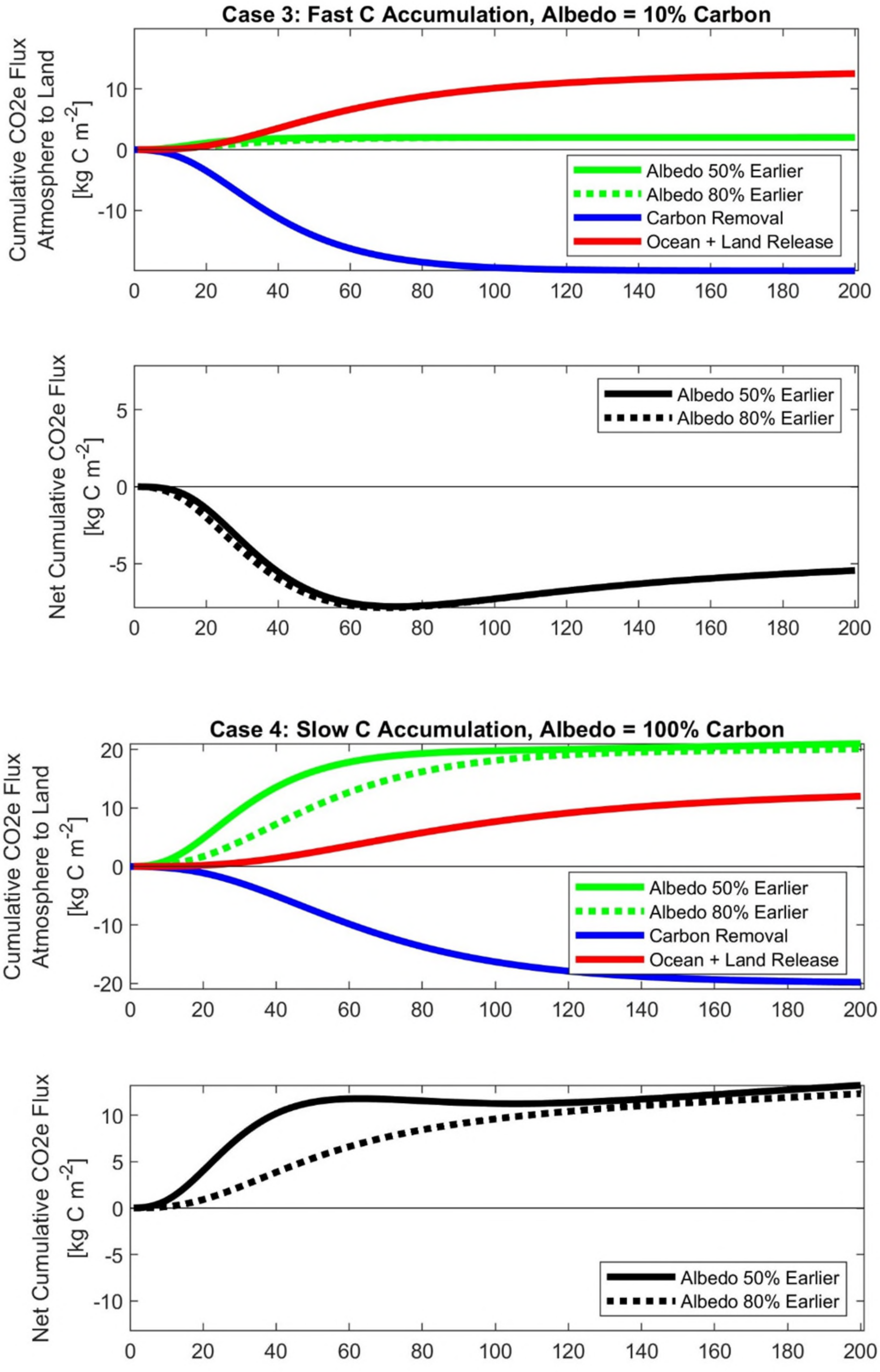
Case 3: Fast C Accumulation, Albedo = 10% Carbon
Cumulative CO2e Flux Atmosphere to Land [kg C m-2]
Albedo 50% Earlier
Albedo 80% Earlier
Carbon Removal
Ocean + Land Release
Net Cumulative CO2e Flux [kg C m-2]
Albedo 50% Earlier
Albedo 80% Earlier
Case 4: Slow C Accumulation, Albedo = 100% Carbon
Cumulative CO2e Flux Atmosphere to Land [kg C m-2]
Albedo 50% Earlier
Albedo 80% Earlier
Carbon Removal
Ocean + Land Release
Net Cumulative CO2e Flux [kg C m-2]
Albedo 50% Earlier
Albedo 80% Earlier

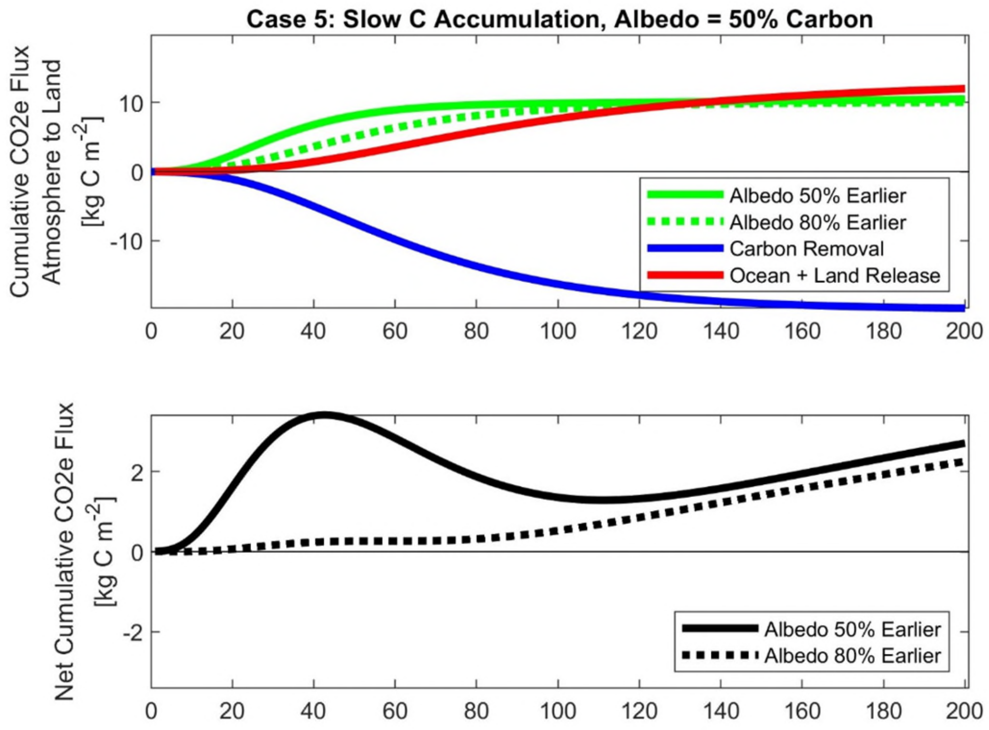

Case 5: Slow C Accumulation, Albedo = 50% Carbon
Cumulative CO2e Flux Atmosphere to Land [kg C m-2]
Albedo 50% Earlier
Albedo 80% Earlier
Carbon Removal
Ocean + Land Release
Net Cumulative CO2e Flux [kg C m-2]
Albedo 50% Earlier
Albedo 80% Earlier

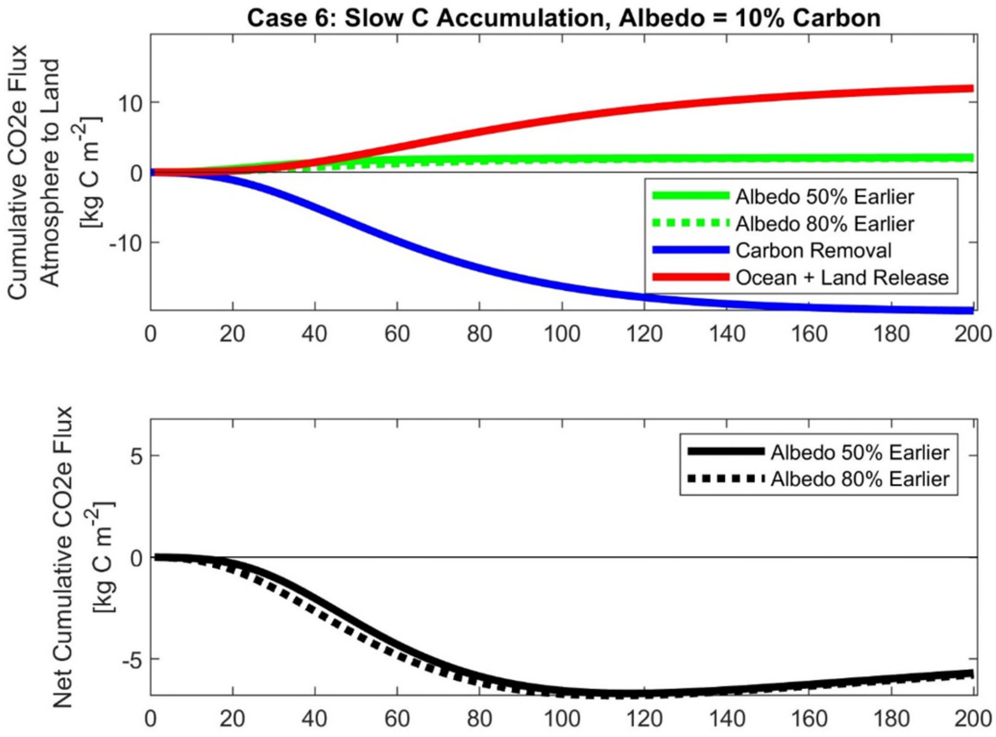

Case 6: Slow C Accumulation, Albedo = 10% Carbon
Cumulative CO2e Flux Atmosphere to Land [kg C m-2]
Albedo 50% Earlier
Albedo 80% Earlier
Carbon Removal
Ocean + Land Release
Net Cumulative CO2e Flux [kg C m-2]
Albedo 50% Earlier
Albedo 80% Earlier

Figure S8. Histogram of the spatial proximity used to establish the most likely (a) open land or (b) forest class, expressed as percent of each biome's area. From light to dark, areas are assigned either the present land cover or the land cover that is dominant within the respective ecoregion within a 0.01° grid (≤0.01°), within the 0.025° or 0.05° grids (≤0.05°), within the 0.1° grid (≤0.01°), within the 0.25° or 0.5° grids (≤0.5°), within the 1° grid (≤1°), within the 2.5° or 5° grids (≤5°), within the 10° grid (≤10°), the dominant land cover over the entire ecoregion, the dominant land cover within their respective biome, climate-zone and region, or finally the dominant land cover for their respective biome world-wide (see Methods). We provide the data for these histograms (see Data Availability).

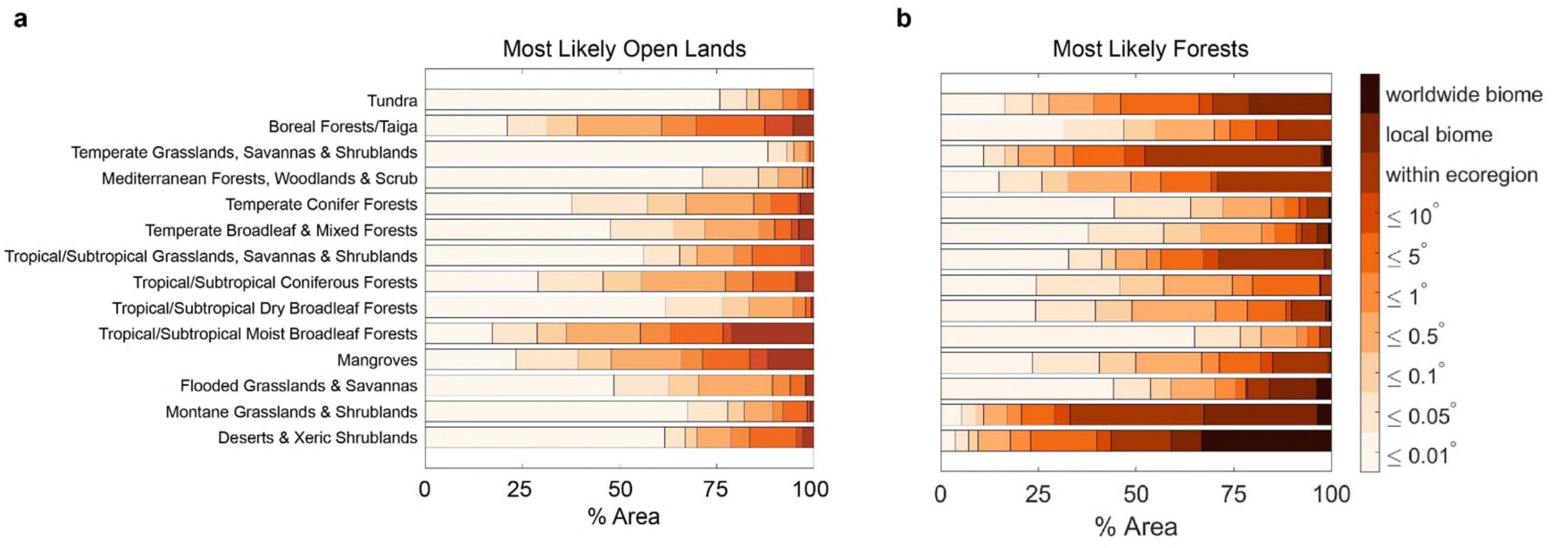

Figure S9. Scenarios of forest carbon accumulation over time. Code for recreating these figures is available (see Code Availability).

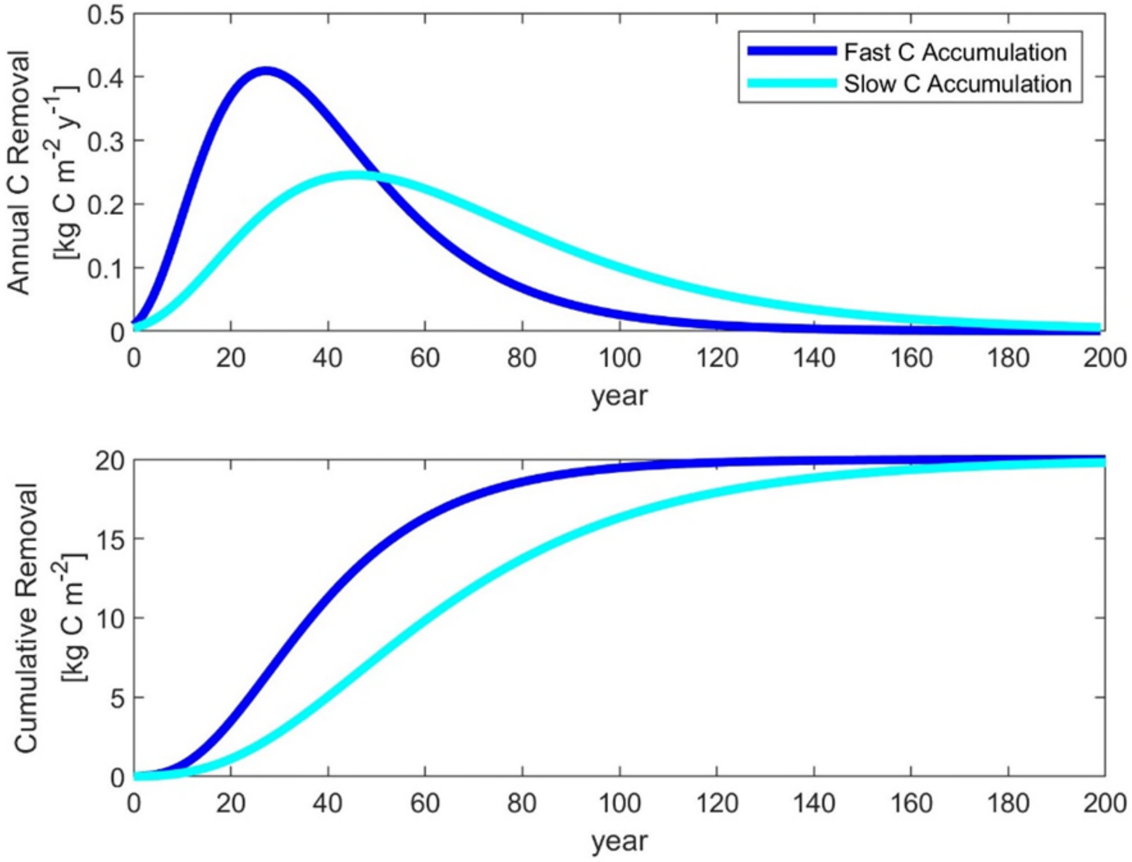

Supplementary References

1. Belward, A. E. *The IGBP-DIS Global 1 Km Land Cover Data Set "DISCover" - Proposal and Implementation Plans*. 63 (1996).

2. Loveland, T. R. & Belward, A. S. The IGBP-DIS global 1km land cover data set, DISCover: First results. *Int J Remote Sens* **18**, 3289–3295 (1997).

3. Griscom, B. W. *et al.* Natural climate solutions. *P Natl Acad Sci Usa* **114**, 11645–11650 (2017).

4. Bastin, J.-F. *et al.* The global tree restoration potential. *Science* **365**, 76–79 (2019).

5. Walker, W. S. *et al.* The global potential for increased storage of carbon on land. *Proc National Acad Sci* **119**, e2111312119 (2022).

6. Block, K. & Mauritsen, T. Forcing and feedback in the MPI-ESM-LR coupled model under abruptly quadrupled CO2. *J Adv Model Earth Sy* **5**, 676–691 (2013).

7. Bright, R. M. & O'Halloran, T. L. Developing a monthly radiative kernel for surface albedo change from satellite climatologies of Earth's shortwave radiation budget: CACK v1.0. *Geosci Model Dev* **12**, 3975–3990 (2019).

8. Pendergrass, A. G., Conley, A. & Vitt, F. M. Surface and top-of-atmosphere radiative feedback kernels for CESM-CAM5. *Earth Syst Sci Data* **10**, 317–324 (2018).

9. Shell, K. M., Kiehl, J. T. & Shields, C. A. Using the Radiative Kernel Technique to Calculate Climate Feedbacks in NCAR's Community Atmospheric Model. *J Climate* **21**, 2269–2282 (2008).

10. Smith, C. J. *et al.* Understanding Rapid Adjustments to Diverse Forcing Agents. *Geophys Res Lett* **45**, 12,023-12,031 (2018).

11. Smith, C. J., Kramer, R. J. & Sima, A. The HadGEM3-GA7.1 radiative kernel: the importance of a well-resolved stratosphere. *Earth Syst Sci Data* **12**, 2157–2168 (2020).

12. Deng, L., Shangguan, Z. & Sweeney, S. "Grain for Green" driven land use change and carbon sequestration on the Loess Plateau, China. *Sci Rep-uk* **4**, 7039 (2014).

13. Crowther, T. W. *et al.* Restor: Transparency and connectivity for the global environmental movement. *One Earth* **5**, 476–481 (2022).

14. Santoro, M. & Cartus, O. ESA Biomass Climate Change Initiative (Biomass_cci): Global datasets of forest above-ground biomass for the years 2010, 2017 and 2018, v2. (2021).
